# P-873. Retrospective Review of Adherence to System Based Skin and Soft Tissue Infection Antimicrobial Guidelines at Hospital Admission

**DOI:** 10.1093/ofid/ofaf695.1081

**Published:** 2026-01-11

**Authors:** Bailey Burnette, Rebecca Reece, Catessa A Howard, Jesse M Thompson, Jordan R Burnette

**Affiliations:** WVU Medicine, Morgantown, WV; West Virginia University, Morgantown, West Virginia; West Virginia University Medicine, Morgantown, West Virginia; West Virginia University, Morgantown, West Virginia; U.S. Department of Veterans Affairs, Morgantown, West Virginia

## Abstract

**Background:**

In literature, institutional antimicrobial guideline adherence is poor and often antimicrobials prescribed are broader than required. Through our own previous institutional review of community acquired pneumonia guideline adherence, we found guideline adherence was only 59% with most being overtreated.This prompted a review of our skin and soft tissue (SSTI) guideline adherence.

**Methods:**

A retrospective chart review was completed on all adult patients admitted to an internal medicine or hospitalist team at WVU hospitals with admission diagnosis within the category of SSTI between January through June 2023. Both demographic and clinical characteristics were collected in addition to outcomes of interest, including empiric antimicrobial choices and adherence to our institutional SSTI guidelines. Data was collected and analyzed via WVU REDCap.

**Results:**

Our cohort included 95 patients with admission diagnosis within SSTI category, but 14 patients were excluded due to being transfers. Demographics showed 63% male and median age of 60 years. 42% received antibiotics in the preceding 30 days, and 82.4% of those antibiotics were for a SSTI. Comorbidities identified included: type II diabetes (33.3%), active malignancy (3.7%), renal disease (17.3%), liver disease (6.2%), chronic lymphedema (12.3%), and immunosuppression (14.8%) (Figure 1). Of the total cohort, 58.0% had risk factors for MRSA. Allergies to antibiotic(s) were documented in 35.8%. Adherence to institutional guidelines was 33.3%, with most non-adherent cases being due to undertreatment (53.0%) (Figure 2). Of the 43 undertreatment cases, 39.5% received previous antibiotics, 37.2% met SIRS criteria, and 11.6% had both. The most common antibiotics prescribed across the total cohort were vancomycin (66.7%), cefepime (42.0%), and ampicillin/sulbactam (13.6%).

**Conclusion:**

With only 33.3% adherence to SSTI guidelines, it shows a need for further education/accessibility to internal guidelines. Given 79.6% of non-adherent cases were due to undertreatment, it questions whether pseudomonal/anaerobic coverage should be recommended in all patients meeting criteria for severe cellulitis. This study may serve as reason to review our institutional SSTI guidelines and/or reviewing the criteria for severe cellulitis.

**Disclosures:**

All Authors: No reported disclosures

